# Capabilities of Double-Resonance LPG and SPR Methods for Hypersensitive Detection of SARS-CoV-2 Structural Proteins: A Comparative Study

**DOI:** 10.3390/bios13030318

**Published:** 2023-02-24

**Authors:** Tinko Eftimov, Petia Genova-Kalou, Georgi Dyankov, Wojtek J. Bock, Vihar Mankov, Sanaz Shoar Ghaffari, Petar Veselinov, Alla Arapova, Somayeh Makouei

**Affiliations:** 1 Photonics Research Center, Université du Québec en Outaouais, Rue 101 St-Jean Bosco, Gatineau, QC J8X 3G5, Canada; 2 Central Laboratory of Applied Physics, Bulgarian Academy of Sciences, 61 Sanct Peterburg Blvd., 4000 Plovdiv, Bulgaria; 3 National Center of Infectious and Parasitic Diseases, 44A “Gen. Stoletov” Blvd., 1233 Sofia, Bulgaria; 4 Institute of Optical Materials and Technologies “Acad. J. Malinowski” (IOMT), Bulgarian Academy of Sciences (BAS), 109 “Acad. G. Bonchev” Str., 1113 Sofia, Bulgaria; 5 Faculty of Electrical and Computer Engineering, University of Tabriz, Tabriz 5166616471, Iran

**Keywords:** biosensors, SARS-CoV-2, long period grating, surface plasmon resonance, structural proteins

## Abstract

The danger of the emergence of new viral diseases and their rapid spread demands apparatuses for continuous rapid monitoring in real time. This requires the creation of new bioanalytical methods that overcome the shortcomings of existing ones and are applicable for point-of-care diagnostics. For this purpose, a variety of biosensors have been developed and tested in proof-of-concept studies, but none of them have been introduced for commercial use so far. Given the importance of the problem, in this study, long-period grating (LPG) and surface plasmon resonance (SPR) biosensors, based on antibody detection, were examined, and their capabilities for SARS-CoV-2 structural proteins detection were established. Supersensitive detections of structural proteins in the order of several femtomoles were achieved by the LPG method, while the SPR method demonstrated a sensitivity of about one hundred femtomoles. The studied biosensors are compatible in sensitivity with ELISA and rapid antigen tests but, in contrast, they are quantitative, which makes them applicable for acute SARS-CoV-2 infection detection, especially during the early stages of viral replication.

## 1. Introduction

### 1.1. Comparison of SARS-CoV-2 Clinical Detection Techniques

In light of the continuously emerging global pandemic of coronavirus disease 2019 (COVID-19) [1], caused by severe acute respiratory syndrome coronavirus 2 (SARS-CoV-2) [2], rapid, highly sensitive, and specific testing is urgently required for the effective management of viral infection [3]. The detection limit is very important because of the relatively low viral concentration in patient samples. Currently, there are several commercially based methods for detection of SARS-CoV-2, which can be divided into three categories: (1) molecular genetic testing such as nucleic acid detection by reverse-transcription polymerase chain reaction (RT-qPCR) (recognized as the gold standard) [4] or other amplification methods [5] and whole-genome sequencing [6] (2) rapid tests focusing on the detection of presence of virus or its fragment, responsible for triggering part of our immune system’s defense response (point-of-care antigen-based methods) [7]; and (3) serological tests detecting the presence of antibodies against SARS-CoV-2 (enzyme-linked immunosorbent assay (ELISA)) [8]. However, the sensitivity and specificity of laboratory techniques for the detection of SARS-CoV-2 depend on some factors: (1) the postinfection day on which the test is performed [9]; (2) type of clinical specimens [10]; (3) the collection procedure and handling of the samples [11], and the concentration of virus in the specimen [12]; (4) the detection sensitivity limit [13]; (5) the time of analysis and the need for specialized laboratory equipment, reagents, and trained staff [14].

Despite its many limitations, the RT-qPCR technique remains the standard based on the amplification of several targeted genomic regions (i.e., *ORF1b*, RNA-dependent RNA polymerase-encoding RdRp gene, and viral nucleocapsid N-, spike S-, or envelope E-genes), which improve its sensitivity (98%) and specificity (up to 95%), especially within the first 5 days after exposure [15]. The detection limit of RT-qPCR kits varies from 0.3 to 100 copies /µL [16].

Rapid SARS-CoV-2 antigen tests are less sensitive than RT-PCR but are now available worldwide and can be directly applied at the point of care (POC) and yield results in 15 min [17]. Most antigen kits target SARS-CoV-2 N-protein labeled with colloidal gold (CG) [18]. They demonstrate ≥80% sensitivity and ≥97% specificity in the diagnosis of SARS-CoV-2 infection [19]. Anti-SARS-CoV-2 IgM antibodies are the first response of the immune system to infection, but IgG is detectable 7–10 days after the infection and can persist for as long as several months [20]. Serological tests, including ELISA, are based on the detection of antigen–antibody interaction [21]. N-protein-based ELISA provides better sensitivity than S-protein based ELISA, because the N protein is the main protein recognized by our immune system and possesses longer persistence than other structural proteins of SARS-CoV-2 [22,23]. Anti-SARS-CoV-2 N-antibodies have been detected with high specificity in the early stage of infection [24], but ELISA assay requires an analysis time of several hours and specific laboratory equipment [25]. The limit of detection of SARS-CoV-2 N protein is 50 pg/mL [26].

### 1.2. Biosensors for Rapid Detection of SARS-CoV-2

In view of the above, rapid and sensitive diagnostic methods for the detection of SARS-CoV-2 that combine all advantages of the other tests and avoid their drawbacks is of growing importance. Many recent studies have considered that biosensors-based techniques can enhance sensitivity and lower the limit of detection [27,28]. Numerous types of sensors have been successfully tested and have demonstrated their feasibility. However, until now, no biosensor has been approved for clinical practice and translated into commercial use.

Refractive-index-based sensors, such as long-period grating (LPG) and surface plasmon resonance (SPR) platforms, are suitable for biosensing applications. LPG, exhibiting double resonance (DR LPG) and microcavity in-fiber Mach–Zehnder interferometers (μIMZI), have been shown to be suitable for bacteria [29] and virus [30] detection. Additionally, reflective-type LPGs (RT-LPGs) were successfully used to detect drug-resistant bacteria [31]. DR LPGs around the turn-around point (TAP) in reflection mode were also proposed as refractive index sensors [32]. Depending on how close the LPGs are to the TAP, their sensitivities to the surrounding refractive index (SRI) changes can vary between about 500 nm/r.i.u at larger split to over 3000 nm/r.i.u. close to the turn around point (TAP) for water-based solutions with a surrounding refractive index (SRI) from 1.333 to 1.37. These sensitivities can be increased by coating the LPG with nanolayers of Al_2_O_3_, diamond-like carbon (DLC) [33], SiN_x_ [34], or TaO_2_ [35]. The latter has shown the highest sensitivity of 11,500 nm/r.i.u. in the 1.335–1.345 r.i.u. range. Recently, phase-shifted LPGs [36] and μIMZI [37] successfully detected spike proteins of the SARS-CoV-2 virus. Comprehensive reviews of SPR-based sensors for SARS-CoV-2 describe recent achievements and limitations [38,39].

The versatility of SPR sensors was highlighted [40] in terms of the applicability of multiphoton and nonlinear processes. The comparative analysis showed the advantage of SPR biosensors related to multiphoton processes. In [41], information was provided on the recent results obtained with SPR detection of SARS-CoV-2. Special attention was paid to the analysis of multilayered structures that are capable of supporting SPR, showing potential for virus detection.

Both DR LPGs and SPR can use specific anti-SARS-CoV-2 monoclonal/polyclonal antibodies as biorecognition elements to detect structural viral antigens, such as the S and N proteins. Therefore, this kind of biosensors may be clinically useful and able to detect acute SARS-CoV-2 infection, especially during the early stages of viral replication.

In this paper, we report the detection of SARS-CoV-2 structural proteins by DR LPG and SPR and comment on the capabilities of both methods. We also evaluated the detection capabilities of the two methods with those of the adopted ELISA laboratory techniques and rapid antigen tests for the detection of SARS-CoV-2.

## 2. Reagents and Materials

All the chemicals and reagents used were of analytical grade.

### 2.1. Structural Proteins

We used the following SARS-CoV-2-specific structural proteins for the evaluation of bimolecular interactions.

#### 2.1.1. SARS-CoV-2 Spike S1 Subunit Protein

The SARS-CoV-2 spike S1 subunit protein fused to a C-terminal poly-histidine (6 x histidine) tag with a tri-amino acid linker (molecular weight (Mw) ~ 123 kDa) were purchased from InvivoGen Company, San Diego, CA, USA. Stock solutions for the experiments were prepared at an initial concentration of 100 µg/mL in endotoxin and nuclease-free water (DEPC-treated water, ThermoFisher Scientific, Invitrogen, Waltham, MA, USA). Aliquots were prepared and stored at −20 °C until use. Working concentrations were propagated in DEPC-treated water in the concentration range of 8–800 fmol.

#### 2.1.2. SARS-CoV-2 Nucleocapsid Protein

SARS-CoV-2 nucleocapsid protein fused to an IgG1 Fc tag with a TEV (tobacco etch virus) sequence linker (Mw ~ 79 kDa) was purchased from InvivoGen Company, San Diego, CA, USA. Stock solutions were prepared at an initial concentration of 100 µg/mL in DEPC-treated water. Aliquots were stored at −2 °C until use. Working concentrations of the stock solution were dissolved in DEPC-treated water in the concentration range of 13–700 fmol.

### 2.2. Antibodies

#### 2.2.1. Anti-SARS-CoV-1/2 NP Antibody

Anti-SARS-CoV-1/2 NP antibody clone 1C7C7 ZooMAb^®^ mouse monoclonal (mAb) (Sigma-Aldrich Inc., St. Louis, MO, USA) (Mw ~ 46 kDa) was prepared at a working concentration of 2.5 µg/mL in DEPC-treated water and then stored at −20 °C until use.

#### 2.2.2. SARS-CoV-2 Nucleocapsid Polyclonal Antibody

SARS-CoV-2 nucleocapsid polyclonal antibody (pAb), IgG, and rabbit polyclonal were purchased from Sigma-Aldrich Inc., St. Louis, MO, USA. The solutions were prepared as described in Section 2.1 and Section 2.2.

### 2.3. Reagents for ELISA

#### 2.3.1. SARS-CoV-2 Antigen ELISA Kit: N Proteins

We used a semiquantitative enzyme immunoassay kit (SARS-CoV-2 Antigen ELISA) for in vitro detection of nucleocapsid protein of SARS-CoV-2 (Euroimmun Medizinische Labordiagnostica AG, Lübeck, Germany). The experiments were performed according to the test instructions.

#### 2.3.2. SARS-CoV-2 Antigen ELISA Kit: S Proteins

We used a qualitative human SARS-CoV-2 (COVID-19) spike protein antigen ELISA kit (Krishgen BioSystems, Mumbai, India). The experiments were performed according to the test instructions.

#### 2.3.3. COVID-19 Antigen Rapid Test

The test was purchased from Acro Biotech Inc., Rancho Cucamonga, CA, USA and was performed according to the instructions.

## 3. Optical Platforms and Functionalization for Virus-Sensing Applications

### 3.1. SPR Platforms

**Figure 1:**
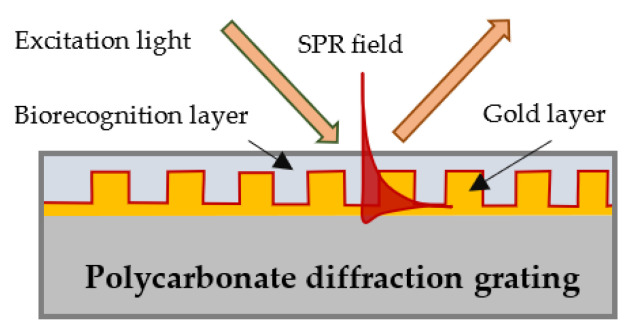
Surface plasmon wave excitation and a biochip-gilded diffraction grating with a layer of immobilized biomolecules.

In our SPR platform, the plasmon wave was excited on the surface of a gilded diffraction grating, as schematically shown in Figure 1. SPR conditions were fulfilled for a P-polarized light beam that illuminated the grating at an incidence angle of about 35 degrees. Typically, the resonance was excited in the range of 695–705 nm for a bare grating having 80 nm grooves at a distance of 1.55 µm from one another [42]. Our SPR biochip represented the grating covered with a biorecognition layer of mono/polyclonal antibodies of certain thickness, as shown in Figure 1.

As a diffraction grating, we used a grooved polycarbonate substrate of a CD-R disc with dimensions 15 × 20 mm. Because we noticed that the curvature of the grooves affected the measurement accuracy, we only used substrates with an equal curvature. The substrates were covered with about 110 ± 10 nm gold film coating obtained by vacuum evaporation.

**Figure 2:**
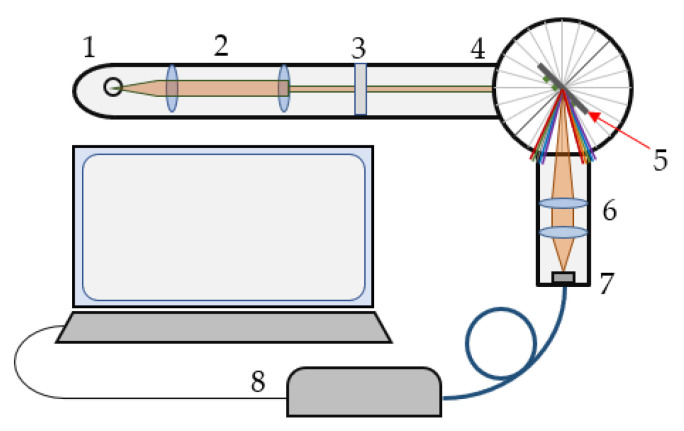
Optical set-up: 1—tungsten lamp, 2—collimator, 3—polarizer, 4—goniometer, 5—biochip, 6,7—objectives, and 8—spectrometer.

The optical set-up used for SPR experiments is shown in Figure 2. A spectral readout for SPR observation was used. White light was collimated and passed through a polarizer that controlled the polarization of the incident light. After reflection from the biochip, the light was focused on a fiber bundle, which transported the light to the spectrometer. To precisely control the angle of incidence, all elements were incorporated into a goniometer. The spectrum was observed at zero-order diffraction.

### 3.2. Double-Resonance Long-Period Gratings (DR LPG)

#### 3.2.1. Turn-Around Point (TAP) and Double Resonance (DR) LPGs

**Figure 3:**
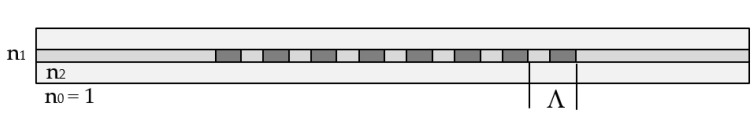
Schematic representation of a long-period grating (LPG).

Long-period grating (LPG) is a structure consisting of a periodic modulation of the refractive index and/or the geometric dimensions of the core and cladding of a stripped optical fiber. These modulations cause coupling between the fundamental LP_01_ core mode of the effective refractive index *n*_01_ and an LP_0p_ higher-order cladding mode of the glass/air waveguide. For a certain period Λ of these modulations (Figure 3), the resonance of the periodic intermodal coupling and the power transfer from the LP_01_ core to LP_0p_ cladding mode occurs at a center wavelength.

(1) $$\lambda_{c}=\Delta n_{eff}\Lambda \;\;\;\Delta n_{eff}=n_{01}-n_{0p}$$

**Figure 4:**
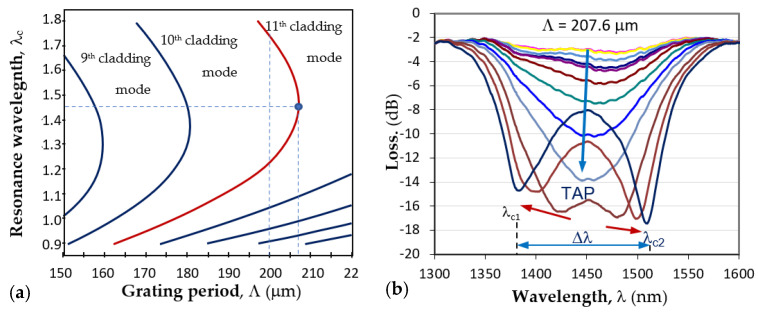
LPG around turning point: (**a**) dispersion dependence and the TAPs of photosensitive fiber PS1250/1500 (Fibercore) for different cladding modes; (**b**) the splitting of a TAP LPG and the transformation into a double resonance grating for the 11th cladding mode.

At this wavelength, the minimum of the transmission spectrum is observed because LP_0p_ cladding mode is leaky in the nonstripped fiber section. As seen from (1), the center wavelength is proportional to the period, which is a unique geometrical quantity that does not depend on the wavelength. However, because of dispersion, for a given grating period, Λ the effective refractive index difference, Δn_eff_ can have two different values, Δn_eff,1_ and Δn_eff,2_, at two different wavelengths, due to which we have two resonance wavelengths, λ_c1_ and λ_c2_, for the same grating period, as shown in Figure 4a. Thus, (1) becomes:

(2) $$\lambda_{c1}=\Delta n_{eff,1}\Lambda \;\;\;\lambda_{c2}=\Delta n_{eff,2}\Lambda$$

with SRI being the basis for label-free biosensors.

The point where λ_c1_ = λ_c2_ is noted in Figure 4a as the turn-around point (TAP), to which corresponds a definite grating period Λ_0p_ for mode LP_0p_. For the 11th cladding mode LP_0,11_ of the photosensitive fiber PS1250/1500 (Fibercore), Λ_0,11_ ≈ 207.7μm. Figure 4b shows the evolution of the LPG spectrum with period Λ at the turning point. The spectrum deepened and split as either temperature or surrounding refractive index increased, which leads to the increase in the spectral separation Δλ = λ_c2_ − λ_c1_. The change in Δλ with SRI provides the basis for label-free biosensors.

#### 3.2.2. DR LPG Fabrication and Calibration Procedure

First, the double-resonance LPGs were fabricated using a SYNRAD-pulsed CO_2_ laser (Synrad Inc., Mukilteo, WA, USA; λ = 10.6 μm, *f* = 20 kHz, at 13.1% to 14% of maximum power, focal length of the scanning head was 10 cm, and spot size was about 100 μm). A Super K Compact white light source (NKT Photonics, Birkerød, Denmark) and a Yokogawa AQ 6370C (Yokogawa Test&Measurement Corporation, Tokyo, Japan) optical spectrum analyzer (OSA) were used to monitor the formation of the grating spectrum during the inscription process of the grating, with *Λ* = 207.6 μm and *N* from 235 to 250. A section with an *l*_0_ = 10 cm length of photosensitive PS 1250/1500 (Fibercore) was spliced between 1 m of lead-in and lead-out SMF-28E single-mode fiber. A weight (*m* = 4 g) was attached to the fiber to guarantee repeatability in the longitudinal fiber strain.

Second, after the inscription, the LPG was immersed in water to check if the desired double-resonance spectrum was achieved.

Third, the grating was placed in a 10% solution of HF acid, and the split was reduced to a desired position around the turning point.

Fourth, after a grating was tuned closer to the TAP, its sensitivity to the SRI around water was measured at 23 °C. The spectral separation, Δλ = λ_c2_ − λ_c1_, between the minima as a function of the SRI *n* in the 1.33–1.35 range was found to be in the form

(3) $$\Delta \lambda =S_{n}n+A$$

In (3), *A* is a constant, and *S*_n_ is the SRI sensitivity. Four of the fabricated DR LPGs with consecutive fabrication numbers P102, P111, P099, and P112 were further functionalized using the procedure described in Section 3.3. Table 1 summarizes the individually measured SRI sensitivities, *S*_n_, and the type of antibody used to functionalize it using a pulsed-laser deposition technique.

### 3.3. Functionalization of the Platforms

The immobilization of biorecognition molecules (ligand) on DR LPG and SPR biosensing transducers is a demanding procedure. The conventional approach involves using a suitable mediating matrix of molecules between the surface of the chip and the biorecognition molecules, which preserves the bioactivity. This matrix, however, generates a nonspecific response. Our approach was different: to immobilize the ligands without using a built-in matrix and use the matrix-assisted pulsed laser evaporation (MAPLE) method instead. The main idea of the MAPLE technique is that the matrix, in which the ligand is dissolved, absorbs the laser power, so that the decomposition of the recognition molecules is avoided. In a previous publication [43], we showed that this technique provides the deposition of intact molecules, as well as high-accuracy and -sensitivity detection [44].

For biorecognition molecules’ deposition, we used a frozen target consisting of 31 pM/mL mAb/pAb dissolved in DEPC water. This concentration was established after many experiments, as the use of MAPLE technology requires a detection sensitivity tradeoff with accuracy. Details regarding the MAPLE technique and the parameters of the immobilization procedure can be found in [43].

The deposition of ligands was simultaneously performed on DR LPG structures with sensitivities measured and on gilded diffraction gratings. The transducers were placed on a vacuum camera in groups of eight and were subsequently functionalized by pulsed-laser deposition of mAb/pAb antibody using the MAPLE technique.

## 4. Experiment, Results, and Analysis

### 4.1. Measurement Procedures

#### 4.1.1. DR LPG Measurement Procedure

Having laser-deposited the mono/polyclonal antibodies upon the grating surfaces, the measurement procedure was performed as follows:

1. The functionalized grating was placed in the measurement set-up using the same weight and at the same temperature as during the calibration measurement.
2. The spectral separation was measured in air and in water immediately after immersion in water and 5 min later, which is referred to as Δλ_0_. The purpose of the 5 min waiting period was to allow for the grating to reach thermodynamic equilibrium in the liquid.
3. The spectra for each concentration, starting from the lowest to the highest, were consecutively measured. Measurements were recorded immediately after the insertion into a particular concentration, after 2.5 min, and at 5 min.
4. After the measurement at the highest concentration was performed, the spectra in water and in air were remeasured.
5. The spectral separation Δλ = λ_c2_ − λ_c1_ for each measurement was determined.
6. Next, the change in the spectral separation at a given concentration Δλ_i_ with respect to that in water Δλ_0_ was defined as
  (4) $$\delta \lambda_{i}=\Delta \lambda_{i}-\Delta \lambda_{0}$$
7. Because the accumulation of the detected protein increased the refractive index upon the surface of the grating, the spectral changes, δλ_i_, were converted into refractive index changes, δ*n*_i_, for the corresponding concentration by taking into account the sensitivity, *S*_n_, of the particular grating. From (3) and (4), it follows that
  (5) $$\delta n_{i}=\frac{\delta \lambda_{i}}{S_{n}}$$
8. Ultimately, the dependence *δn*_i_(*C*_i_) was plotted for each protein and functionalization (mAb and pAb).

**Figure 5:**
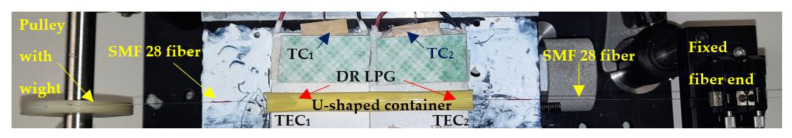
Experimental jig: the DR LPG spliced between two SMF 28 fibers was placed in a U-shaped container (yellow), which was placed on two thermoelectric coolers, TEC_1_ and TEC_2_, mounted on a heat sink covered with thermoconductive paste (white) and upon which were fixed two thermocouples, TC_1_ and TC_2_.

A halogen lamp was used as a white light source, with an AVASPEC-NIR256-1.7-EVO IR Avantes (Apeldoorn, The Netherlands) optical fiber spectrometer sensitive in the 890 to 1750 nm wavelength range. One end of the grating was fixed, while the other was under tension using the same weight (4 g) as for the calibration measurements. The U-shaped container for the solutions under test was mounted on a heat sink whose temperature was maintained at 23 °C by means of thermoelectric coolers (TEC_1_ and TEC_2_), whose temperature was controlled by thermocouples (TC_1_ and TC_2_). A photo of the described arrangement is shown in Figure 5.

#### 4.1.2. SPR Measurement Procedure

The measurement procedure was as follows:

1. After the gold layer was deposited on the polycarbonate substrate, the plasmon resonance was measured at six different points of the diffraction grating surface to eliminate the influence of the grooves’ curvature on the resonance spectral shift.
2. After gilded diffraction gratings were functionalized, the measurement was performed at the same points to evaluate the quality of the ligand layer. The spectral position of the resonances at each point was taken as a reference, against which the shift due to the antibody–antigen interaction was considered.
3. The SPR chips were incubated for 20 min in N- and S-protein solutions of different concentrations at room temperature. Special attention was paid to ensure the uniform coverage of the entire surface of the biochip. Then biochips were washed with deionized water (<2 µS/cm), after which the liquid phase was removed by centrifugation.
4. Plasmon resonances were measured at the same six points on the biochip surface, and resonance wavelength shift was estimated as the difference from the reference resonance. Then, corresponding displacement average values and the absolute measurement errors were determined.

### 4.2. Results

#### 4.2.1. DR LPG Results

**Figure 6:**
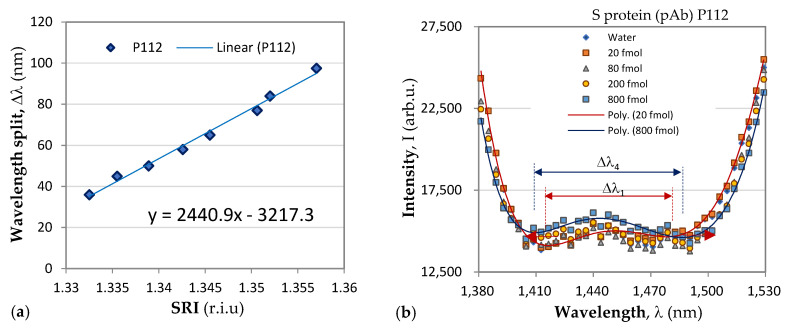
Change in spectrum under four different concentrations of S protein of the P112 grating functionalized with pAb.

**Figure 7:**
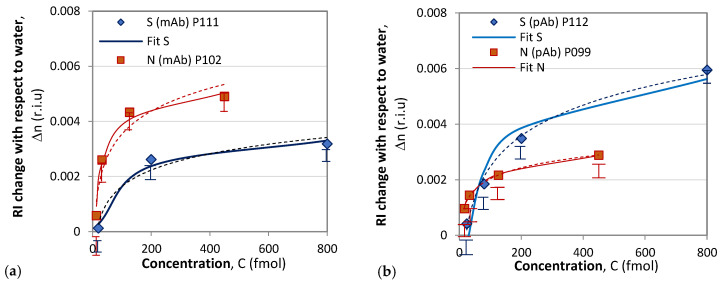
Composite results of responses to S and N proteins: (**a**) with mAb functionalization; (**b**) with pAb functionalization.

Following the above-described procedure, we functionalized gratings P102 and P111 with mAb, and P099 and P112 with pAb. An example of the sensitivity calibration and the response to S protein of P112 is shown in Figure 6. Figure 6b shows the spectral changes in the DR LPG under the four different concentrations into femtomoles (fmol) listed in the legend. The sixth-degree polynomial fit of the DR LPG spectral responses for the lowest (*C*_1_ = 20 fmol) and the highest (*C*_4_ = 800 fmol) concentrations in the 1380 to 1530 nm spectral range are shown in Figure 6b. Additionally, the corresponding spectral separations, Δλ_1_ and Δλ_4_, as found from the polynomial fit are indicated. Figure 7 shows the results of the *δn*(*C*) dependencies obtained with the four gratings, subdivided into two groups according to the functionalization: mAb (Figure 7a) and pAb (Figure 7b). As shown by these graphs, the SRI caused by the protein accumulation upon the grating surface initially quickly rose and was then followed by a slower increase.

The *δn*(*C*) dependence could be sufficiently well-fitted by two types of dependences. The first was by a power function and the second was by a logarithmic dependence as:

(6) $$\delta n(C)=\delta n_{\infty }\left(1-aC^{-\alpha }\right)$$

(7) $$\delta n(C)=A\ln\left(\frac{C}{C_{0}}\right)$$

In (6), *δn*_∞_ is the saturation level of the SRI change as the concentration approaches infinity. The power law approximation in Equation (6) thus implies a saturation level of the SRI around the grating. The logarithmic fit from Equation (7) implies an infinite growth of the SRI change with the accumulation of detected proteins. Table 2 summarizes the values of the parameters *δn*_∞_, *a*, and *α* in the power law approximation (6), while Table 3 summarizes parameters A and B of the logarithmic approximation. The coefficient of determination *R*^2^ for each approximation and grating suggested that the power law was a better approximation, except for P099.

**Table 2:** Power law fitting parameters for the response to protein concentration.

DR LPG	P102	P111	P099	P112
Functionalization	mAb	mAb	pAb	pAb
*δn*_∞_	0.0069	0.0069	0.01	0.01
*a*	1.58	2.0145	1.1	2.250
*α*	0.166	0.329	0.071	0.245
*R* ^2^	0.9762	0.9773	0.9576	0.9986

**Table 3:** Logarithmic fitting parameters for the response to protein concentration.

DR LPG	P102	P111	P099	P112
Functionalization	mAb	mAb	pAb	pAb
*A*	0.0012	0.0009	0.0006	0.0015
*C*_0_	0.18888	0.07765	0.36788	0.05322
*R* ^2^	0.921	0.9576	0.9981	0.9825

The accuracy of the spectral shift measurements was 0.5 nm, which transferred into an uncertainty in the *δn* measurement ranging from 0.00029 for P102 to 0.0002 for P112, which is indicated by the experimental points in Figure 6. It is worth noting the differences between the results reported here and those in [36]. In [36], a phase-shifted LPGs was used, and no sensitivities were reported. Additionally, the observed spectral shift was about 0.7 mm when detecting S-protein solutions with concentrations over an eight-order range. In our study, we used of DR LPGs around the TAP, whose sensitivity was the highest, and the observed a spectral shift was 14.5 nm (Table 1) when the concentration changed by two orders of magnitude, which showed the higher sensitivity of DR LPG. Because individual sensitivities differ, the results are presented in terms of refractive index changes, unlike in [36], where the responses are presented in nanometers. In our measurements, a fiber-optic spectrometer with lower resolution was used, and wavelength shifts were determined from the minima of a polynomial fit. The minimum concentrations detected in [36] are three orders of magnitude lower than those reported here (1 ng/mL ≈ 8 fmol); however, the uncertainty in the measurements was about 30% versus less than 10% in the present work. Because the IR spectrometer used in our case is a compact and lower-cost instrument, our results are promising for laboratory applications. The average wavelength shift of 11 nm observed in this study for a concentration of 800 fmol (≈100 ng/mL) is somewhat better than the wavelength shift of 9.5 nm for 300 ng/mL measured by a μIMZI [37]. The μIMZI used exhibited a much higher sensitivity of 14491 nm/r.i.u. However, the interaction length was only 50 μm versus 48 mm in our case.

#### 4.2.2. SPR Measurement Results

The results of the SPR measurement procedure are summarized in Figure 7. We observed pronounced mAb–N protein interaction for concentrations above 126 fmol. For an N-protein concentration of 126 fmol, the measured spectral displacement was 3.5 nm, which is above the limit of detection (LOD) accounting for measurement error (in this case, 0.47 nm). The LOD was evaluated by considering the accuracy of the spectrometer as well as the accuracy of the goniometer for setting up the angle of light incidence. The probability of reliably measuring concentrations lower than 126 fmol was small because the SPR displacement was compatible with the LOD, and the measurement error increased.

However, the results achieved can be rated as excellent: they are better than or comparable to those reported in the literature, where sophisticated methods for assisting plasmonic resonance were applied. In [45], the author reported an LOD of 0.22 pmol in protein detection with a photothermal-enhanced plasmonic biosensor. The LOD achieved in [46] was 85 fmol in N-protein detection with a nanoparticle-enhanced SPR. A record sensitivity was reported in [47] of 12 fg/mL in the detection of S protein by SPR excited in a multilayer structure including graphene. The good sensitivity achieved by our SPR biosensor is due in part to the direct ligand immobilization method performed by MAPLE technology.

**Figure 8:**
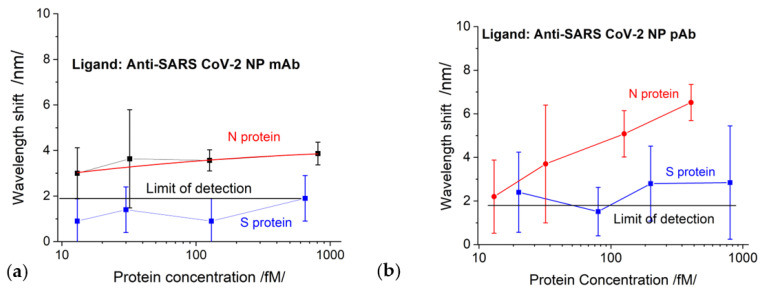
The displacement of SPR as a function of protein concentrations for chips functionalized with: (**a**) SARS-CoV-2 mAb; (**b**) SARS-CoV-2 pAb.

The interactions of mAb–S proteins generated displacements below the LOD, as illustrated in Figure 8a, which was partly due to the direct immobilization of the mAb and to its specificity.

S-protein binding to pAb generated a signal slightly above the detection limit but within the measurement error zone, as shown in Figure 8b. The N-protein detection by pAb (red curve in Figure 8b) was better expressed than the detection by mAb; however, the LOD was the same: 126 fmol.

We would like to specifically note that the concentration dependences established by the SPR method were observed only in the indicated concentration range of the structural proteins and for the indicated thickness of the gold grating coating.

### 4.3. Comparative Analysis: DR LPG vs. SPR

The comparison of the results of DR LPG and SPR clearly showed that the sensitivity and accuracy of DR LPG measurement was several times better. As shown in Figure 6a, reliable detection was achieved for an N-protein concentration of 13 fmol, while for the SPR method, reliable detection was achieved with a concentration of 126 fmol. The recorded signal that resulted from the interaction with S protein (Figure 6a blue curve) is surprising for a specific monoclonal anti-SARS-CoV-2 mAb against N protein (however, several times lower than that of the N protein), which could be explained by the higher sensitivity of the method. At the same time, the SPR detection did not show interactions with SARS-CoV-2 S protein (Figure 8a).

To some extent, the highest sensitivity of the DR LPG method could explain the result illustrated in Figure 6b: the interaction with the S protein generated a stronger signal than that with the N protein, despite of the fact that polyclonal antibody is specific for the N-protein. However, it should be noted that the specificity of polyclonal antibodies is lower than monoclonal antibodies, which have one epitope to join with a specific viral antigen.

It is most likely that this is a background signal appearing, not from the nonspecific binding of the S-protein to pAb, but due to accumulation (concentration) of a large amount of the protein around the epitopes on the surface of the antibody. This is probably why the signal was above the LOD for SPR detection (blue curve in Figure 8b). However, the lower sensitivity of the SPR method did not allow such an expressive difference, as in the case of DR LPG.

### 4.4. Comparative Analysis: DR LPG/SPR vs. ELISA/Rapid Antigen Test

**Figure 9:**
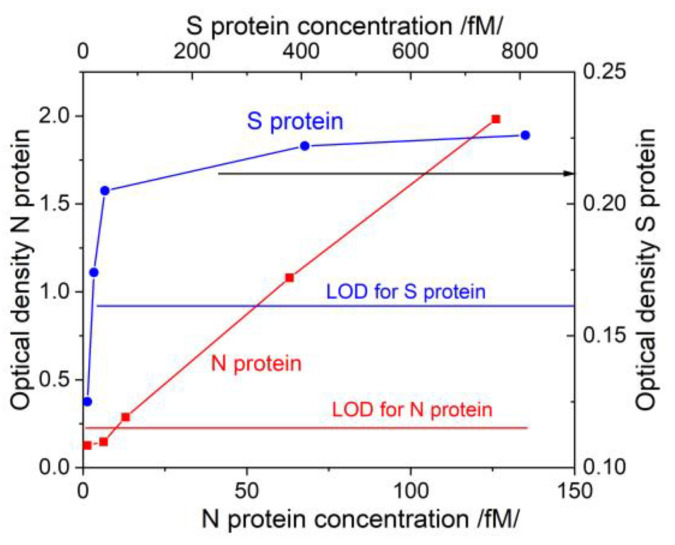
Concentration dependences for N/S proteins obtained via ELISA.

It is very important to compare the capabilities of the two studied methods with those used in clinical practice. For this purpose, we determined the concentration dependences for the structural proteins used in our study with the ELISA method. Standard antigen kits for SARS-CoV-2 structural N/S proteins were used. Figure 9 shows the results in terms of optical density (OD).

The LODs of the ELISA tests were determined according to the implementation instructions. The LOD for N protein was 10 fmol. The results recorded with the DR LPG method (Figure 7) were absolutely compatible with those of ELISA. We draw the attention to the fact that the antibody used for the ELISA test is not officially reported and probably not the same as that used in our study. In this sense, the results cannot be fully compared.

**Figure 10:**
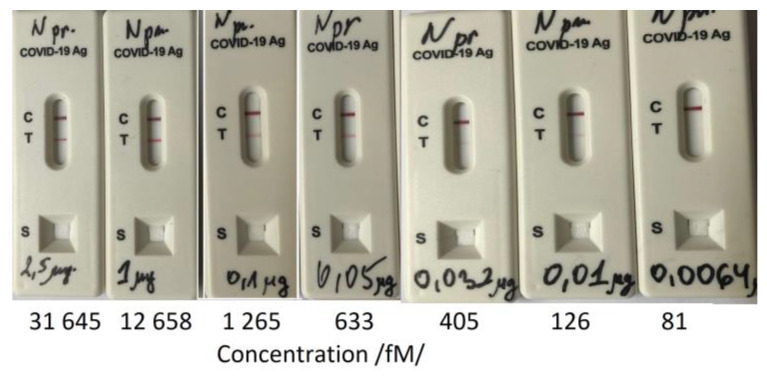
Limit of Detection (LOD) determination of a rapid antigen test.

In general, rapid antigen tests are designed to detect N proteins. We used one to determine its LOD by treatment with N-protein solutions of different concentrations. The results are shown in Figure 10.

The LOD as slightly lower than 126 fmol. The SPR measurement was 126 fmol as the first reliably measured concentration, which fully corresponded to the LOD of the antigen test. The LOD of the DR LPG method was one order lower. Table 4 presents a summary of the achieved sensitivities.

**Table 4:** Comparison of established limits of detection for DR LPG, SPR and clinical tests.

Ligand		N-mAb	N-pAb
Detected Proteins		N-Protein	N Protein
Biosensor	DR LPG	13 fmol	20 fmol
	SPR	126 fmol	126 fmol
Clinical test	ELISA	10 fmol	--
	Rapid antigen test	126 fmol	--

We would like to point out that the antibodies and antigens used in our study are commercially available. This makes it possible to compare our results with those of validated tests. The advantage of the DR LPG biosensor is that it is much faster than ELISA at comparable sensitivities.

The SPR sensor showed a sensitivity comparable to the best reported to date [45,46]. As mentioned, this is partly due to MAPLE immobilization. Another factor is the very good signal-to-noise ratio achieved owing to the sharp SPR dip: the average FWHM was 18 nm. The sharp SPR resonance was due to the precise control of the parameters of vacuum deposition of gold layer and the angle between the plane of incidence and the grating grooves. The above-mentioned influence of the grooves’ curvature on the measurement accuracy is related to the deviation from the perpendicular direction of the plane of incidence to the grooves. This also requires a high degree of polarization of the p-polarized incidence light. For this purpose, a polarizer with an extinction coefficient of 5 × 10^5^ was used. These are the factors determining the LOD.

It is though that the multilayered structures in the Kretschmann configuration significantly improve the sensitivity and accuracy of detection [47]. This approach has not been considered for grating structures, but given the results reported here, a multilayer structure is promising. Research in this topic is planned. We would like to pay particular attention to the method of functionalization.

MAPLE technology allows the deposition of nanostructured layers with uniform thickness and very good adhesion. Moreover, the technology is feasible, not labor-intensive, and provides excellent specificity.

## 5. Conclusions

Comparing the capabilities of the DR LPG and SPR methods in terms of sensitivity and accuracy showed the advantage of the former: it detected 13 fmol while SPR detected 126 fmol of N protein. LPG measurement accuracy was also better. The reason is that the spectral shifts of each grating were considered with respect to deionized water. It is vital to have a stable reference before antibody–antigen events start. In the case of SPR measurements, the baseline is the reference resonance of the functionalized grating, measured with the some error that should be considered in the detection of the analyte. This, however, gives an advantage to the SPR method: it can be applied as a point-of-care test.

The comparison of the two methods with clinically used ones showed that the DR LPG method fully corresponds to ELISA, while the SPR method ensures sensitivity and accuracy similar to those of rapid antigen tests. It should be noted that the studied methods are quantitative, which makes them suitable for following the disease evolution during acute SARS-CoV-2 infection, especially during the early stages of viral replication, which can be clinically useful.

## Figures and Tables

**Table 1:** Sensitivity *S*_n_ to SRI and type of functionalization of the DR LPGs used in the experiments.

DR LPG	P102	P111	P099	P112
Number of periods, N	235	235	240	235
SRI sensitivity *S*_n_ (nm/r.i.u)	1732.8	2327.2	2081.4	2440.9
Functionalization	mAb	mAb	pAb	pAb
Maximum concentration (fmol)	450	800	450	800
Maximum shift (nm)	8.5	7.5	6	14.5

## Data Availability

No data publicly available.
